# STAR CAREGIVERS: IMPLEMENTATION OF AN EVIDENCE-BASED PROGRAM IN REAL-WORLD SETTINGS

**DOI:** 10.1093/geroni/igad104.2012

**Published:** 2023-12-21

**Authors:** Susan McCurry, Robert Penfold, Michele Karel

## Abstract

Evidence-based non-pharmacological community programs to improve care of older adults with dementia are growing. However, many programs have historically been tested in research studies that fail to reach medically underserved populations or communities with unique service needs. STAR-Caregivers (STAR-C, also known as STAR-Community Consultants) is an evidence-based psychosocial support and skill training program designed to teach family caregivers how to identify and increase pleasant events, improve communication, and use behavioral problem-solving skills to reduce the problems experienced by their family member with dementia while improving quality of life for both persons in the caregiver-care receiver dyad. Since its original randomized controlled trial in 2005, STAR-C has been translated in a variety of real-world settings, including in Area Agencies on Aging throughout Oregon and Washington state. This symposium describes three recent new translation efforts: (1) implementation of STAR-C as a virtual training program (STAR-VTF) for caregivers of persons with dementia who are part of the Kaiser Permanente Washington health care system; (2) cultural adaptation of STAR-VTF for Latino caregivers, and (3) incorporation of STAR-C into a largely rural community care setting in Illinois during the COVID-19 pandemic using Amazon Echo technology to conduct telehealth community dyad STAR-C sessions and connect clients to supplemental services. Each presenter will discuss the dementia care needs in their respective populations, the challenges encountered in translation of STAR-C to serve target caregiving dyads, and lessons learned regarding treatment effectiveness and program sustainability.

